# Modeling Intervention Scenarios During Potential Foot-and-Mouth Disease Outbreaks Within U.S. Beef Feedlots

**DOI:** 10.3389/fvets.2021.559785

**Published:** 2021-02-16

**Authors:** Aurelio H. Cabezas, Michael W. Sanderson, Victoriya V. Volkova

**Affiliations:** ^1^Department of Diagnostic Medicine and Pathobiology, College of Veterinary Medicine, Kansas State University, Manhattan, KS, United States; ^2^Center for Outcomes Research and Epidemiology, College of Veterinary Medicine, Kansas State University, Manhattan, KS, United States

**Keywords:** foot-and-mouth disease, meta-population model, beef feedlot, beef cattle, intervention strategies, cattle depopulation

## Abstract

Foot-and-mouth disease (FMD) is a highly contagious disease of livestock and has severely affected livestock industries during the past two decades in previously FMD-free countries. The disease was eliminated in North America in 1953 but remains a threat for re-introduction. Approximately 44% of the on-feed beef cattle in the U.S. are concentrated in feedlots <32,000 heads, but little information is available on dynamics of FMD in large feedlots. Therefore, there is a need to explore possible management and intervention strategies that might be implemented during potential FMD outbreaks on feedlots. We used a within home-pen stochastic susceptible-latent-infectious-recovered (SLIR) FMD dynamics model nested in a meta-population model of home-pens in a feedlot. The combinatory model was previously developed to simulate foot-and-mouth disease virus (FMDv) transmission within U.S. beef feedlots. We evaluated three intervention strategies initiated on the day of FMD detection: stopping movements of cattle between home-pens and hospital-pen(s) (NH), barrier depopulation combined with NH (NH-BD), and targeted depopulation of at-risk home-pens combined with NH (NH-TD). Depopulation rates investigated ranged from 500 to 4,000 cattle per day. We evaluated the projected effectiveness of interventions by comparing them with the no-intervention FMD dynamics in the feedlot. We modeled a small-size (4,000 cattle), medium-size (12,000 cattle), and large-size (24,000 cattle) feedlots. Implementation of NH delayed the outbreak progression, but it did not prevent infection of the entire feedlot. Implementation of NH-BD resulted in depopulation of 50% of cattle in small- and medium-size feedlots, and 25% in large-size feedlots, but the intervention prevented infection of the entire feedlot in 40% of simulated outbreaks in medium-size feedlots, and in 8% in large-size feedlots. Implementation of NH-TD resulted in depopulation of up to 50% of cattle in small-size feedlots, 75% in medium-size feedlots, and 25% in large-size feedlots, but rarely prevented infection of the entire feedlot. Number of hospital-pens in the feedlot was shown to weakly impact the success of NH-TD. Overall, the results suggest that stopping cattle movements between the home-pens and hospital-pens, without or with barrier or targeted cattle depopulation, would not be highly effective to interrupt FMDv transmission within a feedlot.

## Introduction

Foot-and-mouth disease (FMD) is a highly contagious disease that has affected several non-endemic countries in the past 20 years such as the United Kingdom, Japan, Uruguay, Argentina, the Netherlands, and France (1–7). In North America, the last outbreaks occurred in 1929 in the United States, 1952 in Canada, and 1946 in Mexico; FMD was eventually eliminated in North America in 1953 (8, 9). Nonetheless, there remains a threat of FMD re-introduction to the U.S. with animals infected or animal products contaminated with the foot-and-mouth disease virus (FMDv). The survival and infectivity of FMDv in fomites and excretions, and aspects of the spread of FMDv has been previously reviewed (10–13). An FMD outbreak in the U.S. would cause catastrophic economic consequences for the livestock and associated industries, as has been previously suggested by modeling studies (14–16).

At any given time the U.S. has ~13 million cattle on feed distributed in over 30,000 feedlots across the 48 states (17). Approximately 1% of those feedlots have a one-time capacity equal or greater than 32,000 head; however, they contain ~44% of the cattle on-feed population in the country (17). The main strategies used to control FMD during the course of an outbreak in non-endemic countries have been: livestock movement bans, depopulation of infected and susceptible animals in affected and at-risk areas, sanitary/biosecurity measures, surveillance zones, and emergency vaccination (2–5, 18–21). To our knowledge, there are no studies that evaluate the effectiveness of on-farm (within-feedlot) intervention strategies during FMD outbreaks in large concentrated livestock operations such as beef cattle feedlots. The large concentration of cattle in this type of operation might represent a challenge to the success of any of the control strategies mentioned above. For this reason, there is a need to investigate possible management and intervention strategies that might be implemented during potential FMD outbreaks in large feedlots.

Some authors have used models to simulate potential between-farm FMDv transmission within the contiguous U.S., and to evaluate different intervention strategies such as livestock movement bans, depopulation, and vaccination (22–26). These models do not focus on within-farm transmission dynamics, projecting which is necessary to assess on-farm management and control strategies in large compartmentalized feedlots. Others have used alternative methods to evaluate the potential use of control strategies at the farm level. For instance, the feasibility of cattle depopulation within a large feedlot has been described by McReynolds and Sanderson (27). They used a Delphi survey and facilitated expert discussion to investigate the complete depopulation of a large feedlot considering effectiveness, animal and human welfare, public perception, and availability of needed supplies. The study concluded that humane and timely depopulation of a large feedlot would be difficult. Given the difficulty of depopulation of large U.S. beef feedlots, alternative FMD control strategies may be required. In this study, we evaluated the projected impacts on FMD outbreak progression in a feedlot of several alternative intervention strategies. We used a mathematical model previously developed to simulate the FMDv transmission and clinical manifestation dynamics within U.S. beef feedlots. We investigate the projected effectiveness of intervention strategies such as within-feedlot cattle movement ban, and a barrier or targeted depopulation of subpopulations of the cattle after FMD detection in the feedlot.

## Materials and Methods

### Within-Farm FMD Dynamics Model and Feedlot Layouts

We used a previously published and fully described model by Cabezas et al. (28). The FMD infection and clinical disease dynamics within each home-pen and between home-pens are represented in Equations 1–7 and the model parameters are described in Table 1. Briefly, we modeled beef finishing cattle in five different feedlot layouts (see Table 2, and Supplementary Figures 1–5). The housing system most commonly represented in U.S. beef feedlots is cattle housed within pens (home-pens) with metal fences that allow cattle in contiguous home-pens to have direct nose to nose contact through fence-lines. These feedlots are open air with no roof and dirt floors. Cattle are monitored daily be pen riders who enter the pen and systematically ride through looking for sick cattle. Cattle from different home-pens can have directed contact at the hospital pen if they are pulled from their home-pens to receive treatment. In addition, direct contact between cattle from different home-pens might occur in the drovers alleys while cattle are pulled from home-pens to the hospital pen. Contiguous home-pens may also share a water trough. In each home-pen, we implemented a modified stochastic SLIR model with three I compartments of subclinical infectious one, subclinical infectious two, and clinical infectious cattle. The home-pens were nested in a home-pen meta-population in the feedlot. Two levels of FMDv transmission were modeled: within a home-pen and between home-pens. Within home-pen transmission was modeled via direct contact assuming homogeneous cattle mixing inside the home-pen. Between home-pen transmission was modeled via direct contact of cattle in hospital-pen(s), fence-line direct contact of cattle in contiguous home-pens, by pen-riders moving between home-pens, waterborne through drinking water troughs shared by home-pens, and airborne. More details about the model formulation and between home-pen transmission routes modeled can be found in Cabezas et al. (28). Transmission via cattle direct contact in the alleys of the feedlot, and via contaminated feed were not modeled. Simulations started with a proportion of FMD-latent cattle in an index home-pen located centrally within the feedlot.

**Table 1:** Definitions and values of parameters used in modeling potential foot-and-mouth disease transmission, infection, and clinical manifestation dynamics on U.S. beef cattle feedlots.

**Parameter**	**Definition (units)**	**Mean value and distribution**	**References^a^**
**WITHIN A HOME-PEN**			
*lat_initial*	Initial proportion of latent cattle in the index-pen	0.05, Vector (0.005, 0.105, 0.020)	Assumed
*β_*wp*_*	Beta transmission parameter for virus transmission via direct animal contact in a home-pen (animal^−1^ day^−1^)	0.026, Triangular (0.020, 0.026, 0.031)	Derived from (29)
*lat*	Duration of latent period (days)	3.2, Weibull (α 1.782, β 3.974)	(30)
*sub*	Duration of subclinical period (days)	2.0, Gamma (α 1.222, β 1.672)	(30)
*inf*	Duration of infectious period (days)	4.0, Gamma (α 3.969, β 1.107)	(30)
*cli*	Duration of clinical period (days)	7.5, Fixed	(31)
*cliinf*	Duration of clinical infectious period (days)	(inf-sub) in each model simulation	
*clinon_inf*	Duration of clinical non-infectious period (days)	(cli-clininf) in each model simulation	
δ	Rate of progression to subclinical infectious 1 status (day^−1^)	1/lat	
θ	Rate of progression to subclinical infectious 2 status (day^−1^)	1/(sub/2)	
ε	Rate of progression to clinical infectious status (day^−1^)	1/(sub/2)	
γ	Rate of recovery from being infectious (day^−1^)	1/cliinf	
τ	Rate of recovery from clinical disease after recovering from being infectious (day^−1^)	1/clinon_inf	
υ	Proportion of home-pens with cattle just placed in the feedlot (dmnl)	0.20	Feedlot expert opinion
π	Morbidity rate for bovine respiratory disease (BRD) during the first 30 days since cattle placement in the feedlot	0.162, Vector (0.050, 0.300, 0.050)	(32)
ρ	Morbidity rate for other production diseases during the 200 days since cattle placement in the feedlot	0.1280, fixed	(32)
*brdtrt*	Probability for an animal with BRD to be pulled to a hospital-pen for treatment during the disease course (dmnl)	0.8750, fixed	(32)
*endtrt*	Probability for an animal with other than BRD production diseases to be pulled to a hospital-pen for treatment during the disease course (dmnl)	0.6908, fixed	(32)
φ_*t* = 1to 30_	Per-animal pull rate from a home-pen to hospital-pen due to BRD and other production diseases during the first 30 days since cattle placement in the feedlot (day^−1^)	0.0052	Calculated, $\left(\frac{\pi^{*}brdtrt}{30}\right)+\left(\frac{\rho^{*}endtrt}{200}\right)$
φ_*t* = 31to 200_	Per-animal pull rate from a home-pen to hospital-pen due to production diseases between the days 31 and 200 since cattle placement in the feedlot (day^−1^)	0.0004	Calculated,$\frac{\rho^{*}endtrt}{200}$
ς	Per-animal pull rate from a home-pen to hospital-pen due to clinical FMD (day^−1^)	0.02800	FMD expert opinion
μ	Mortality rate for animals with BRD and other production diseases (endemic infectious diseases and noninfectious diseases) (day^−1^)	Triangular (0.01, 0.03, 0.05)	(32)
ψ	Mortality rate for animals with clinical FMD (day^−1^)	Triangular (0, 0.005, 0.010)	FMD Expert opinion
**Between home-pens**			
In hospital-pen(s)			
*β_*hp*_*	Beta transmission parameter for virus transmission via direct animal contact in a hospital-pen (animal^−1^ day^−1^)	Same as *β_*wp*_*	Derived from (29)
Fence-line
*β_*bp*_*	Beta transmission parameter for virus transmission via fence-line direct animal contact (animal^−1^ day^−1^)	*β_*wp*_*/4	Assumed [*β_*wp*_* derived from (29)]
**ENVIRONMENTAL BY PEN-RIDERS**			
*uri*	Urine volume produced by an animal (L/day)	Uniform (8.8, 22.0)	(13)
*sal*	Saliva volume produced by an animal (L/day)	Uniform (98, 190)	(13)
*fec*	Volume of feces produced by an animal (kg/day)	Uniform (14, 29)	(13)
*uriv*	Virus quantity shed in urine (plaque forming units (PFU)/mL) by an animal in the FMD clinical high infectious status	Uniform (10^2.5^, 10^5.5^)	(13)
*salv*	Virus quantity shed in saliva (PFU/mL) by an animal in the FMD clinical high infectious status	Uniform (10^6^, 10^8^)	(13)
*fecv*	Virus quantity shed in feces (PFU/mL) by an animal in the FMD clinical high infectious status	Uniform (10^2^, 10^4.1^)	(13)
*fsal_env*	Proportion of the cattle daily saliva volume deposited into the home-pen environment (dmnl)	0.3, Vector (0.1, 0.5, 0.1)	Assumed
*fsal_env_floor*	Proportion of *fsal* that lands on the floor (dmnl)	0.33	Assumed
*vir_dec_env*	Virus decay rate in the home-pen floor environment (day^−1^)	0.28, Fixed	(33)
σ	Amount of the home-pen floor materials moved daily to the next home-pen in the row by pen-riders (g/day) (300 g per pen-rider round, two rounds per day)	600, Fixed	Assumed plausible amount carried on horse hooves between pens
*w_pen*	Width of a home-pen (m)	61.0, Fixed	Typical industry value
*l_pen*	Length of a home-pen (m)	75.2, Fixed	Typical industry value
*d_pen*	Depth of a home-pen floor top contaminated with the animal fresh secretions and excretions (m)	0.02, Vector (0.02, 0.05, 0.03)	Expert opinion, typical pen surface loosened by hoof action
*min_oral*	Minimum infective dose of FMDv via oral exposure in cattle (PFU/mL)	10^6^, Fixed	(11)
**Via shared water-troughs**			
*fsal_env_w*	Proportion of *fsal* that lands in the water-trough (dmnl)	(1-*fsal_env_floor*)	Assumed
*vir_dec_w*	Virus decay rate in water (day^−1^)	0.12, Fixed	(33)
*vol_watert*	Volume of the water trough shared between two home-pens (L)	6,000, Fixed	Expert opinion, typical tank size to provide sufficient water reservoir for cattle needs
*min_oral*	Minimum infective dose of FMDv via oral exposure in cattle (PFU/mL)	10^6^, Fixed	(11)
**Airborne**			
α	Power of the exponential function of decay in the airborne transmission with increasing distance between home-pen centroids (dmnl)	−3.5, Fixed	(34)
	Proportion of clinical infectious cattle in a home-pen *k*	Modeled	
*d*_*i, k*_	Scaled distance between centroids of a home-pen *i* and home-pen *k* (*k* is any other home-pen than *i*) (dmnl)	1.0–22.4, Fixed	Euclidean distance between each two home-pen centroids scaled by the shortest Euclidian distance between two home-pen centroids in the feedlot

a *In the reference column: “Assumed” refers to parameter values assigned based on our knowledge/judgement. “Derived from [x]” refers to values that we estimated based on data in the cited references. “[x]” is the reference from which the value was adopted directly. “Expert opinion” refers to values obtained via personal communication with experts in the epidemiology of FMD, and in the feedlot industry*.

*dmnl, indicates the value does not have a unit of measure*.

*PFU, plaque forming units*.

* *Table retrieved from Cabezas et al. (28)*.

**Table 2:** Description of the feedlot size and layout, and the projected duration of the outbreak for the baseline no intervention scenario and NH [(hospital movement restrictions to stop mixing of cattle from different home-pens in the hospital-pen(s) beginning].

**Feedlot modeled**	**Number of home-pens**	**Total number of cattle**	**Number of hospital-pens**	**Intervention scenario modeled^a^**	**Outbreak duration, days (10th, 50th, and 90th percentiles of *n =* 2,000 simulations)^b^**
FS1	20	4,000	1	None	39, 49, 59
				NH	37, 47, 57
FM1	60	12,000	1	None	46, 58, 69
				NH	68, 82, 95
FM2	60	12,000	2	None	61, 74, 89
				NH	69, 82, 95
FL1	120	24,000	2	None	60, 73, 86
				NH	70, 84, 97
FL2	120	24,000	4	None	68, 82, 95
				NH	70, 84, 97

a *None—baseline no intervention scenario (28), NH—hospital movement restrictions to stop mixing of cattle from different home-pens in the hospital-pen(s) after the day of detection*.

b *The projected duration of the outbreak defined as the time in days between the introduction of FMD latent cattle and when the prevalence of infectious individuals within the feedlot is equal to 0*.

The feedlot layouts were: FS1—small-size feedlot with 4,000 cattle distributed in 20 home-pens, and operating with one hospital-pen; FM1—medium-size feedlot with 12,000 cattle distributed in 60 home-pens, and operating with one hospital-pen; FM2—medium-size feedlot with 12,000 cattle distributed in 60 home-pens, and operating with two hospital-pens (30 home-pens per hospital-pen); FL1—large-size feedlot with 24,000 cattle distributed in 120 home-pens, and operating with two hospital-pens (60 home-pens per hospital-pen); and FL2—large size feedlot with 24,000 cattle distributed in 120 home-pens, and operating four hospital-pen (30 home-pens per hospital-pen). In all feedlots modeled, every home-pen had 200 cattle. For those feedlots that operated with more than one hospital-pen, the hospital-pen received cattle from the section of home-pens that was in closest spatial proximity to the hospital-pen. Rows of pens were separated by a feed alley for delivering feed to each pen on one size and a drovers ally for driving cattle to the hospital on the other. No pens shared a feed trough. Pens across a feed or drovers alley could not have nose to nose contact. The detailed model formulation is described by (28). The feedlot layout diagrams are included in Supplementary Figures 1–5.

Susceptible:

(1) $$\begin{array}{l} \frac{\text{dS}}{\text{dt}}=-\beta_{wp}{\text{S(I}}_{1}{\text{+I}}_{2}{\text{+I}}_{3})-\varphi \text{S}-Bin\left(\varphi_{\left(t-1\right)}{\text{S}}_{\left(t-1\right)},\;p\_\text{inf ​​}\_\text{​​}{\text{ hp}}_{l_{\left(t-1\right)}}\right)-\\ \left\{\begin{array}{l} \text{S}\beta_{bp}{({\text{I}}_{1}{\text{+I}}_{\text{2}}{\text{+I}}_{\text{3}}\text{)}}_{j};\;j\text{ present}\\ \text{ 0; otherwise} \end{array}\right\}-\left\{\begin{array}{l} \text{S}\beta_{bp}{\left({\text{I}}_{1}{\text{+I}}_{\text{2}}{\text{+I}}_{\text{3}}\right)}_{h};\;h\text{ present}\\ \text{ 0; otherwise} \end{array}\right\}-\\ \left\{\begin{array}{l} Bin(\text{S},\;0.5);\;j\text{ present, shares water-trough with }i\text{, and FMDv load in 1 L of the water }\geq ID_{50}\text{ per oral}\\ \text{ 0; otherwise} \end{array}\right\}-\\ \;\left\{\begin{array}{l} Bin(\text{S},\;0.5);\;h\text{ present, shares water-trough with }i\text{, and FMDv load in 1 L of the water }\geq ID_{50}\text{ per oral}\\ \text{ 0; otherwise} \end{array}\right\}-\\ \left\{\begin{array}{l} Bin\left[\left(\frac{FMDv\_floor_{j}\times \sigma }{ID_{50}\text{ per oral}}\right),\;0.5\right];\;j\text{ present and }\left(\frac{FMDv\_floor_{j}\times \sigma }{ID_{50}\text{ per oral}}\right)\leq \text{S }\\ \text{ 0; otherwise} \end{array}\right\}-\\ \;\left\{\begin{array}{l} Bin(\text{S},\;p\_air_{i});\;\sum_{k=1}^{n}{\text{I}}_{3}\geq \text{0}\\ \text{ 0; otherwise} \end{array}\right\}-\mu \text{S} \end{array}$$

Latent:

(2) $$\begin{array}{l} \frac{\text{dL}}{\text{dt}}=\beta_{wp}{\text{S(I}}_{1}{\text{+I}}_{2}{\text{+I}}_{3})-\varphi \text{L}+Bin\left(\varphi_{\left(t-1\right)}{\text{S}}_{\left(t-1\right)},\;p\_\text{inf ​​}\_\text{​​}{\text{ hp}}_{l_{\left(t-1\right)}}\right)+\\ \left\{\begin{array}{l} \text{S}\beta_{bp}{({\text{I}}_{1}{\text{+I}}_{\text{2}}{\text{+I}}_{\text{3}}\text{)}}_{j};\;j\text{ present}\\ \text{ 0; otherwise} \end{array}\right\}+\left\{\begin{array}{l} \text{S}\beta_{bp}{\left({\text{I}}_{1}{\text{+I}}_{\text{2}}{\text{+I}}_{\text{3}}\right)}_{h};\;h\text{ present}\\ \text{ 0; otherwise} \end{array}\right\}+\\ \left\{\begin{array}{l} Bin(\text{S},\;0.5);\;j\text{ present, shares water-trough with }i\text{, and FMDv load in 1 L of the water }\geq ID_{50}\text{ per oral}\\ \text{ 0; otherwise} \end{array}\right\}+\\ \;\left\{\begin{array}{l} Bin(\text{S},\;0.5);\;h\text{ present, shares water-trough with }i\text{, and FMDv load in 1 L of the water }\geq ID_{50}\text{ per oral}\\ \text{ 0; otherwise} \end{array}\right\}+\\ \left\{\begin{array}{l} Bin\left[\left(\frac{FMDv\_floor_{j}\times \sigma }{ID_{50}\text{ per oral}}\right),\;0.5\right];\;j\text{ present and }\left(\frac{FMDv\_floor_{j}\times \sigma }{ID_{50}\text{ per oral}}\right)\leq \text{S }\\ \text{ 0; otherwise} \end{array}\right\}+\\ \;\left\{\begin{array}{l} Bin(\text{S},\;p\_air_{i});\;\sum_{k=1}^{n}{\text{I}}_{3}\geq \text{0}\\ \text{ 0; otherwise} \end{array}\right\}-\delta \text{L}-\mu \text{L} \end{array}$$

Subclinical infectious 1:

(3) $$\begin{array}{r} \frac{\text{d}{\text{I}}_{\text{1}}}{\text{dt}}=\delta \text{L}-\theta {\text{I}}_{\text{1}}-\varphi {\text{I}}_{\text{1}}+\varphi_{\left(t-1\right)}{\text{I}}_{{\text{1}}_{\left(t-1\right)}}-\mu {\text{I}}_{1} \end{array}$$

Subclinical infectious 2:

(4) $$\begin{array}{r} \frac{\text{d}{\text{I}}_{\text{2}}}{\text{dt}}=\theta {\text{I}}_{\text{1}}-\varepsilon {\text{I}}_{\text{2}}-\varphi {\text{I}}_{\text{2}}+\varphi_{\left(t-1\right)}{\text{I}}_{{\text{2}}_{\left(t-1\right)}}-\mu {\text{I}}_{\text{2}} \end{array}$$

Clinical infectious:

(5) $$\begin{array}{r} \frac{\text{d}{\text{I}}_{\text{3}}}{\text{dt}}=\varepsilon {\text{I}}_{\text{2}}-\gamma {\text{I}}_{\text{3}}-\left(\varphi +\varsigma \right){\text{I}}_{3}+\left(\varphi_{\left(t-1\right)}+\varsigma \right){\text{I}}_{3_{\left(t-1\right)}}-\left(\mu +\psi \right){\text{I}}_{3} \end{array}$$

Clinical non-infectious

(6) $$\begin{array}{r} \frac{\text{dC}}{\text{dt}}=\gamma {\text{I}}_{\text{3}}-\tau \text{C}-\left(\varphi +\varsigma \right)\text{C}+\left(\varphi_{\left(t-1\right)}+\varsigma \right){\text{C}}_{\left(t-1\right)}-\left(\mu +\psi \right)\text{C} \end{array}$$

Recovered

(7) $$\begin{array}{r} \frac{\text{dR}}{\text{dt}}=\tau \text{C}-\varphi \text{R}+\varphi_{\left(t-1\right)}{\text{R}}_{\left(t-1\right)}\mu \text{R} \end{array}$$

### Intervention Scenarios

The intervention scenarios were applied on the day of FMD detection in the feedlot. The detection was based on observational surveillance of clinical signs by pen-riders which are experienced personnel in feedlots to detect diseased cattle. The detection was assumed to occur when the proportion of FMD clinical cattle in the index home-pen reached a 3% prevalence threshold. The intervention scenarios were applied by modifying the parameter values from the baseline no-intervention scenario models (see Table 1).

Three intervention scenarios were investigated. In the no hospital (NH) scenario, cattle movements from home-pens to the hospital-pens were stopped to prevent mixing of cattle from different home-pens in the hospital-pen(s) beginning on the day of FMD detection. In the no hospital, barrier depopulation scenario (NH-BD), NH was combined with a barrier depopulation. On the day of FMD detection, cattle in the row of home-pens containing the index home-pen and in the home-pens in the adjacent rows were depopulated. No further depopulation was done following completion of the initial barrier depopulation. In the no hospital, targeted depopulation scenario (NH-TD), NH was combined with a trace-back targeted depopulation. On the day of FMD detection, cattle in home-pens that had contact with the hospital-pen(s) within 7 days prior to FMD detection were traced-back, and those home-pens were depopulated. No further depopulation was done following completion of the initial traceback based depopulation. We assumed a baseline depopulation rate of 1,000 cattle per day (or five home-pens each with 200 cattle), but also evaluated the impact of other depopulation rates. For FS1, we also modeled 2,000 cattle per day depopulation rate; for FM1 and FM2, we also modeled 500 and 2,000 cattle per day depopulation rates; and for FL1 and FL2, we also modeled 2,000 and 4,000 cattle per day depopulation rates. See Supplementary Figures 1–5 for a schematic representation of the intervention strategies in the feedlots modeled.

#### NH Intervention Scenario

The daily pulling rate of cattle from home-pens to the hospital-pen (φ) due to endemic infectious and non-infectious diseases was set to 0 to stop all movement to and mixing in the hospital pen starting on the day of FMD detection.

#### NH-BD Intervention Scenario

The barrier depopulation started on the day of FMD detection. The cattle mortality rate was set to 100% in a home-pen on the day of depopulation. The home-pens were depopulated in the inside-out order: the index home-pen, then home-pens in the row where the index home-pen is located, and then home-pens in rows adjacent to the index home-pen row. The number of home-pens depopulated each day was constrained to reflect the assumed maximum daily depopulated rate (head/day) for the feedlot.

#### NH-TD Intervention Scenario

The traceback based target depopulation started on the day of FMD detection. The cattle mortality rate was set to 100% in a home-pen on the day of depopulation. Only home-pens were depopulated that had cattle coming back from the hospital-pens within 7 days of FMD detection. We prioritized depopulation of home-pens based on the spatial location in the feedlots in relation to the index home-pen. Home-pens in the row containing the index home-pen were depopulated first, then home-pens in adjacent rows and so on. The number of home-pens depopulated each day was constrained to reflect the assumed maximum daily depopulated rate (head/day) for the feedlot.

### Outbreak Metrics

We evaluated the following metrics in each feedlot modeled:

#### NH Intervention Scenario

(1) The projected duration of the outbreak for NH compared to the baseline no-intervention scenario defined as the time in days since the introduction of FMD latent cattle until the prevalence of infectious individuals within the feedlot is equal to 0. (2) The projected time to infection of all home-pens for NH compared to the baseline no-intervention scenario. A home-pen was considered infected when at least one animal become FMD latent during the outbreak. (3) The effectiveness of the intervention implemented in preventing FMD spread. We defined effectiveness of the intervention strategies as the percentage of simulations in which FMD transmission was interrupted and no further susceptible cattle in were infected after implementation of the intervention strategy in each feedlot and intervention modeled.

#### NH-BD Intervention Scenario

The effectiveness of the intervention implemented in preventing FMD spread. We defined effectiveness of the intervention strategies as the percentage of simulations in which FMD transmission was interrupted for each feedlot and intervention modeled. Success was defined as an iteration where no further pens were infected following the barrier depopulation.

#### NH-TD Intervention Scenario

The effectiveness of the intervention implemented in preventing FMD spread. We defined effectiveness of the intervention strategies as the percentage of simulations in which FMD transmission was interrupted for each feedlot and intervention modeled. Success was defined as an iteration where at least 1 pen remained uninfected at the end of the outbreak.

The baseline model verification and validation processes were previously conducted by Cabezas et al. (28). We finally compared the results of the intervention strategies to those of the baseline no-intervention scenario (results for the baseline no intervention scenario are reported in Cabezas et al. (28).

### Model and Its Output Statistical Analysis Implementation

The model was implemented in Vensim® PLE Plus Version 6.4a (Ventana Systems Inc., Harvard, MA, USA). The output figures were done in R using the ggplot package and the schematics of the depopulation interventions in Microsoft Office Power Point® 365 ProPlus (Microsoft, Redmond, WA, USA). The statistical analysis of the model outputs was done in STATA® 13 (StataCorp LP, College Station, TX, USA). We provide median and percentiles of the results to best represent some non-normal outcome distributions.

### Sensitivity Analysis

The target parameters included in the sensitivity analysis were the FMD latent, infectious, and subclinical periods, and the beta transmission parameter within the home-pens (see Table 1). We simulated the model for each feedlot size and layout, for a scenario in which FMD latent cattle are introduced in an index home-pen located centrally within the feedlot, and for each intervention scenario. The value of each target parameter was sampled for each of 2,000 Monte Carlo simulations. For each of the other parameters in the model, a single value was used for each of the 2,000 simulations (see Table 1).

For NH, we investigated the effect of the target parameters and the day of FMD detection on the projected duration of the outbreak. Using the outputs of the 2,000 model simulations for the feedlot size and layout, we used the Spearman rank correlation coefficient to estimate the correlation between each of the target parameters and the day of FMD detection, and between the number of home-pens depopulated and the projected outbreak duration. For NH-BD and NH-TD, we investigated the effect of the target parameters along with the number of hospital-pens in the feedlot, the day of FMD detection, and the number of pens depopulated (only in NH-TD) on the effectiveness of the interventions. Using the outputs of the 2,000 model simulations for the feedlot size and layout, we used the Spearman rank correlation coefficient to estimate the association of the target parameters, the day of FMD detection, and the number of home-pens depopulated with the projected outbreak duration. We also estimated the proportion of simulations with uninfected home-pens at the end of the outbreak. For NH-BD, a fixed number of home-pens was depopulated for each feedlot modeled (10 in FS1, and 30 for all others); then, if the remaining home-pens were not infected during the simulations (10 inf FS1, 30 for FM1 and FM2, and 90 for FL1 and FL2), we considered the intervention successful. For NH-TD, the number of home-pens depopulated was variable depending on how many home-pens had contact with the hospital-pen in the 7 days before FMD detection. So, we considered the intervention successful if at least one home-pen remained uninfected at the end of the outbreak. Descriptive statistics for the projected number of depopulated and uninfected home-pens during the simulations were summarized. Finally, we estimated the effect of modeling different depopulation rates on the outcome described above. For FS1, FM1, and FM2 we modeled depopulation rates of 500 and 2,000 cattle per day while we modeled depopulation rates of 2,000 and 4,000 cattle per day for FL1 and FL2.

## Results

### Outbreak Progression and Duration

The projected outbreak duration was only compared between NH and the baseline no intervention scenario in which depopulation was not implemented. The largest variation in the projected duration of the outbreak when NH was implemented was seen in FM1. Implementation of this strategy was found to significantly increase the projected duration of the outbreak when compared to the baseline no intervention scenario (82 days compared to 58 days for the baseline no intervention scenario). For FM2 and FL1 the projected median duration of the outbreak for the baseline no intervention scenario was 73 days, and implementation of NH increased it by 8 (FM2) to 11 days (FL1). For FS1 and FL2, there were no changes in the median projected duration of the outbreak when NH was implemented compared to the baseline no intervention scenario (49 days for FS1 and 84 days for FL2). See Table 2 for more detailed results.

### Time to Infection of All Home-Pens

The projected time to infection was only compared between NH and the baseline no intervention scenario. The projected time to infect all home-pens since FMDv introduction when comparing NH to the baseline no intervention scenario was longest for FM1. All home-pens took a projected median of 22 days to become infected for the no intervention scenario compared to a projected median of 54 days when NH was implemented. The second largest difference was for FL1 in which the projected median time for all home-pens to become infected for the no intervention scenario was 37 days compared to 53 days for NH. For the rest of feedlots modeled the projected median time to infect all home-pens was 40 days for the baseline scenario compared to 54 days for NH (FM2), 46 days for the baseline scenario compared to 54 for NH (FL2), and 15 days for the baseline scenario compared to 18 days for NH (FS1).

### Effectiveness of Different Depopulation Strategies Depending on the Maximum Depopulate Rate for the Feedlot

**Figure 1:**
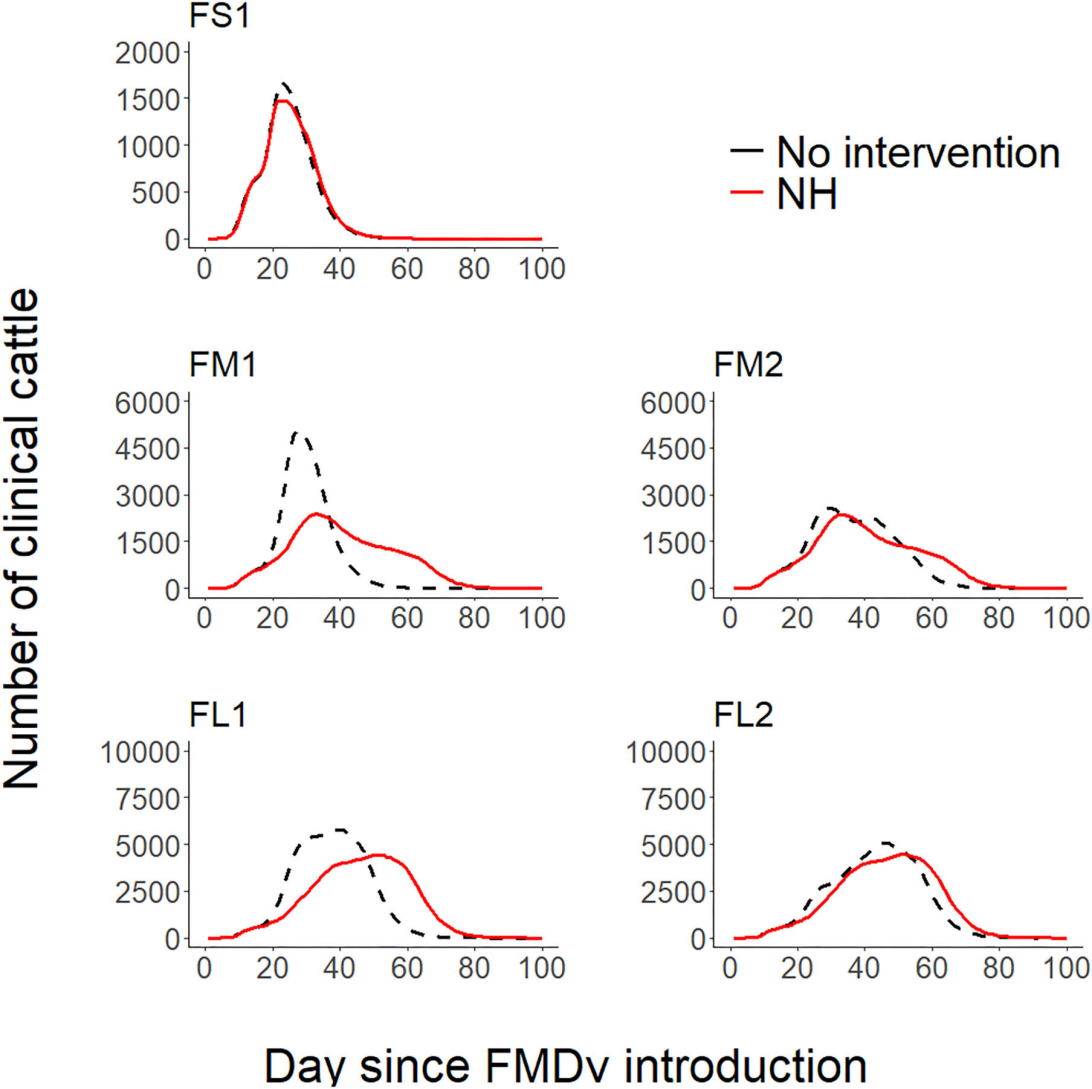
Line plots comparing the projected foot-and-mouth outbreak curves for clinical cattle count (Y-axis) during each day since FMDv introduction (X-axis) between the baseline no intervention scenario (28) and the hospital movement restriction (NH) scenario for each feedlot of size and layout. FS1—small-size feedlot with one hospital-pen, FM1—medium-size feedlot with one hospital-pen, FM2—medium-size feedlot with two hospital-pens, FL1—large-size feedlot with two hospital-pens; and FL2—large-size feedlot with four hospital-pens. The black dashed lines represent the 50th percentile of the distribution of the projected count of clinical cattle across 2,000 simulated outbreaks for the baseline no intervention scenario for each feedlot size and layout (28), red solid lines represent the 50th percentile of the distribution of the projected count of clinical cattle across 2,000 simulated outbreaks for NH—hospital movement restrictions to stop mixing of cattle from different home-pens in the hospital-pen(s) after the day of FMD detection.

**Figure 2:**
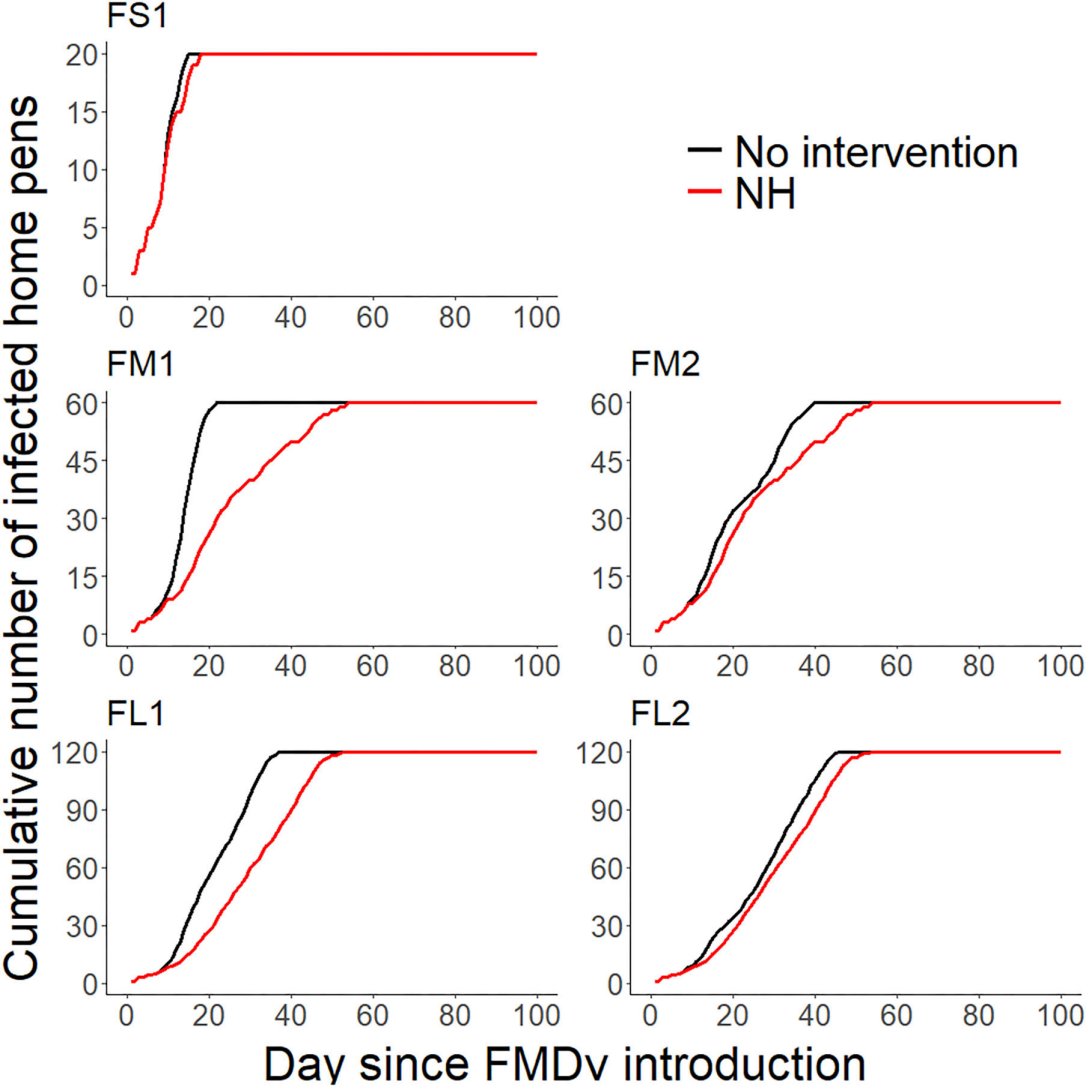
Line plots comparing the projected cumulative number of infected home-pens (Y-axis) during each day since FMDv introduction (X-axis) between the baseline no intervention scenario (28), and the hospital movement restriction (NH) scenario for each feedlot of size and layout. FS1—small-size feedlot with one hospital-pen, FM1—medium-size feedlot with one hospital-pen, FM2—medium-size feedlot with two hospital-pens, FL1—large-size feedlot with two hospital-pens; and FL2—large-size feedlot with four hospital-pens. The black solid lines represent the 50th percentile of the distribution of the projected cumulative number of infected home-pens across 2,000 simulated outbreaks for the baseline no intervention scenario for each feedlot size and layout (28), red solid lines represent the 50th percentile of the distribution of the projected cumulative number of infected home-pens across 2,000 simulated outbreaks for NH—hospital movement restrictions to stop mixing of cattle from different home-pens in the hospital-pen(s) after the day of FMD detection.

Implementation of NH was unsuccessful in preventing FMD infection in all feedlots modeled. NH delayed the projected time to infect the entire population but eventually all cattle were infected in all feedlots modeled (see Figures 1, 2 and Table 2 for more detailed information).

Barrier depopulation (NH-BD) was the intervention with the highest probability of success in interrupting FMD infection resulting in no additional infected home-pens after its implementation (Table 3). For feedlots FM1 and FM2 (30 home-pens depopulated) continued transmission of FMDv infection was interrupted (30 home-pens uninfected following barrier depopulation) in 16% of simulations when depopulation was set at 500 cattle per day and in 38-40% of simulations if 1,000 or 2,000 cattle per day were depopulated. For FL1 and FL2 (30 pens depopulated), continued transmission of FMDv infection was interrupted (90 home-pens uninfected following barrier depopulation) in 41–42% of simulations when 4,000 cattle were depopulated per day; 34–38% of simulations when depopulation was set at 2,000 cattle per day but decreased to only 7–8% when depopulation was set at 1,000 cattle per day. NH-BD (10 home-pens depopulated) was never successful in interrupting FMD transmission in FS1 (no remaining uninfected home-pens) for either depopulation rate modeled (1,000 or 2,000 cattle per day) (see Table 3).

**Table 3:** Projected effectiveness of NH-BD (NH combined with barrier depopulation) and NH-TD (NH combined with targeted depopulation) for the feedlot size and layout and the different depopulation rates modeled, 2000 simulations.

**Feedlot modeled^a^**	**Depopulation rate (cattle per day**	**Percent of simulations successful (%)^b^**	**Number of home-pens depopulated (25th, 50th, 75th percentiles)**	**Percent of successful simulations (%)^c^**	**Number of non-infected home-pens in successful simulations (%)^c^ (25th, 50th, 75th percentiles)**
		**NH-BD**	**NH-TD**	**NH-TD**	**NH-TD**
FS1	1,000	0	10, 11, 11	0	NA^d^
	2,000	0		0	NA
FM1	500	16	42, 43, 45	82	1, 2, 12
	1,000	38		91	2, 18, 18
	2,000	38		94	4, 15, 18
FM2	500	16	21, 25, 29	68	1, 2, 4
	1,000	39		68	1, 2, 4
	2,000	40		70	1, 2, 4
FL1	1,000	7	30, 33, 36	42	1, 1, 1
	2,000	34		46	1, 1, 1
	4,000	41		48	1, 1, 1
FL2	1,000	8	25, 27, 30	46	1, 1, 2
	2,000	39		47	1, 1, 2
	4,000	42		47	1, 1, 2

a *FS1 is a 4,000 cattle feedlot with 1 hospital-pen; FM1 is a 12,000 cattle feedlot with 1 hospital-pen; FM2 is a 12,000 cattle feedlot with 2 hospital-pens; FL1 is a 24,000 feedlot with 2 hospital-pens; and FL2 is a 24,000 cattle feedlot with 4 hospital-pens (in all the layouts n = 200 cattle per home-pen)*.

b *We defined success of NH-BD intervention strategies as the percentage of simulations in which FMD transmission was interrupted for each feedlot and intervention modeled resulting in no remaining infected pens at the end of the barrier depopulation*.

c *We defined effectiveness of NH-TD the intervention strategies as the percentage of simulations in which FMD transmission was interrupted for each feedlot and intervention modeled resulting in at least one uninfected pen at the end of the outbreak*.

d *NA means that there were no successful simulations*.

Targeted depopulation (NH-TD) was less effective compared to NH-BD (Table 3). In no case did targeted depopulation result in no additional infected home-pens following implementation. For this reason, success for NH-TD was defined as any uninfected home-pen at the end of the outbreak. For FM1, if depopulation was set at 500 cattle per day, the percentage of successful simulations was 82%; however, 43 home-pens (72%) were depopulated and a median number of 2 uninfected home-pens remained after the intervention. The percentage of successful simulations increased to over 90% and a median of 15 (25%) and 18 (30%) home-pens remain uninfected when 1,000 or 2,000 cattle were depopulated per day. For FM2, FL1, and FL2, the intervention was poorly effective with rare uninfected home-pens after the intervention with a median number of 25, 33, and 27 home-pens depopulated regardless the number of cattle depopulated per day. NH-TD was never successful in FS1 with a median number of 11 home-pens depopulated in the intervention (see Table 3).

### Sensitivity Analysis

For NH, the sensitivity analysis showed that the duration of the latent period had a strong positive correlation (Spearman rank correlation coefficient ≥0.77 for all feedlots modeled) with the projected duration of the outbreak. The infectious period was moderately positively correlated (Spearman rank correlation coefficient was ~0.28 for FM1, FM2, FL1, and FL2 and 0.47 for FS1) with the projected duration of the outbreak. The subclinical period had a negative weak correlation with the projected duration of the outbreak in all feedlots modeled. The beta transmission parameter did not have a significant association (*p* > 0.05) with the projected duration of the outbreak for any of the feedlots modeled (see Table 4).

**Table 4:** Target parameters investigated for associations with the projected duration of the outbreak after implementation of NH (hospital movement restrictions to stop mixing of cattle from different home-pens in the hospital-pen(s) beginning the day after FMD).

**Target parameter**	**Parameter value distribution^a^**	**Strength of the correlation (Spearman correlation coefficient value) between the model parameter value and the duration of the outbreak for the feedlot of that size and layout^b^**				
		**FS1^c^**	**FM1**	**FM2**	**FL1**	**FL2**
Beta transmission parameter in home-pens (*β_*wp*_*)	Triangular (0.02, 0.026, 0.031)	−0.05	−0.01	0.01	0.01	0.01
Duration of FMD latent period (*lat*) (days)	Weibull (α = 1.782, β = 3.974)	**0.80**	**0.77**	**0.83**	**0.87**	**0.88**
Duration of FMD infectious period (*inf*) (days)	Gamma (α = 3.969, β = 1.107)	**0.47**	**0.28**	**0.29**	**0.28**	**0.28**
Duration of FMD subclinical period (*sub*) (days)	Gamma (α = 1.222, β = 1.672)	**−0.19**	**−0.07**	**−0.07**	**−0.07**	**−0.06**

a *See Table 1 for a more detailed information on target parameters and Supplementary Figures 1–5 for a more detailed information on feedlot layouts*.

b *Bold coefficients indicate p <0.05 for the correlation coefficient between the parameter value and the duration of the outbreak*.

c *FS1 is a 4,000 cattle feedlot with one hospital-pen; FM1 is a 12,000 cattle feedlot with one hospital-pen; FM2 is a 12,000 cattle feedlot with two hospital-pens; FL1 is a 24,000 feedlot with two hospital-pens; and FL2 is a 24,000 cattle feedlot with four hospital-pens (in all the layouts n = 200 cattle per home-pen)*.

We summarized only the results of FM1, FM2, FL1, and FL2 sensitivity analysis for the depopulation scenarios because the interventions were never successful in interrupting FMDv transmission in FS1. For both NH-BD and NH-TD, simulations showed that the duration of FMD stages (latent, infectious, and subclinical period) were weakly correlated with having uninfected home-pens after the interventions for all of the feedlots and for all of the depopulation rates modeled. The number of hospital-pens did not show a significant correlation (*p* > 0.05) with having uninfected home-pens for any depopulation rate after NH-BD was implemented in medium-size feedlots. In contrast, it was found to be significantly (*p* < 0.05) and moderately correlated with having uninfected home-pens after NH-TD was implemented—The higher the depopulation rates the stronger the correlation. In large-size feedlots, for both NH-BD and NH-TD, the number of hospital pens were weakly correlated with having uninfected home-pens (Spearman rank correlation coefficient at most 0.05 for any of the feedlots and for any of the depopulation rates modeled).

The day of FMD detection was moderately negatively correlated with having uninfected home-pens after NH-BD for all feedlots modeled at higher depopulation rates (1,000 and 2,000 cattle per day for medium-size feedlots, and 2,000 and 4,000 cattle per day for large-size feedlots). In contrast, the day of FMD detection was weakly and positively correlated (ranged from 0.5 to 0.24) with having uninfected home-pens after NH-TD for all feedlots and all depopulation rates (see Table 5).

**Table 5:** Target parameters investigated for associations with having uninfected home-pens after implementation of NH-BD (NH was combined with barrier depopulation) and NH-TD (NH was combined with targeted depopulation) for the feedlot size and layouts modeled.

**Target parameters**	**Feedlot size and layout^a^**	**Strength of the correlation (Spearman correlation coefficient value) between the target parameters and presence of uninfected home-pens after implementation of NH-BD and NH-TD for the feedlot of that size and layout^b^**											
		**FM1 & FM2^c^**		**FM1 & FM2^c^**		**FM1 & FM2^c^**		**FL1 & FL2^c^**		**FL1 & FL2^c^**		**FL1 & FL2^c^**	
	Culling Capacity	(500 cattle/day)^d^		(1,000 cattle/day)		(2,000 cattle/day)		(1,000 cattle/day)		(2,000 cattle/day)		(4,000 cattle/day)	
	Control Scenario	NH-BD	NH-TD	NH-BD	NH-TD	NH-BD	NH-TD	NH-BD	NH-TD	NH-BD	NH-TD	NH-BD	NH-TD
Beta transmission parameter in home-pens	Triangular (0.02, 0.026, 0.031)	0.03	0.01	0.01	0.01	0.01	0.02	−0.01	0.01	−0.01	0.01	−0.01	0.01
Duration of FMD latent period (days)	Weibull (α = 1.782, β = 3.974)	**−0.21**	−0.02	**−0.14**	−0.01	**−0.15**	−0.01	**−0.03**	0.01	**−0.10**	0.01	**−0.13**	0.01
Duration of FMD infectious period (days)	Gamma (α = 3.969, β = 1.107)	**−0.17**	**−0.15**	**−0.14**	**−0.14**	**−0.13**	**−0.11**	**−0.07**	**−0.23**	**−0.08**	**−0.23**	**−0.09**	**−0.23**
Duration of FMD subclinical period (days)	Gamma (α = 1.222, β = 1.672)	**0.04**	**0.13**	**0.01**	**0.08**	**0.02**	**0.08**	**0.03**	**0.16**	**0.02**	**0.17**	**0.02**	**0.17**
Number of hospital–pens in the feedlot^e^	Fixed (1 or 2)	0.01	**−0.16**	0.01	**−0.42**	0.01	**−0.52**	**0.02**	**0.05**	**0.05**	**0.01**	**0.02**	**0.01**
Day of FMD detection^f^	Modeled	**−0.23**	**0.11**	**−0.64**	**0.10**	**−0.65**	**0.05**	**−0.26**	**0.21**	**−0.62**	**0.22**	**−0.63**	**0.24**

a *See Table 1 for a more detailed information on target parameters and Supplementary Figures 1–5 in for a more detailed information on feedlots layouts*.

b *Bold coefficients indicate p < 0.05 for the correlation coefficient between the parameter value and the duration of the outbreak*.

c *FM1 is a 12,000 cattle feedlot with one hospital-pen; FM2 is a 12,000 cattle feedlot with two hospital-pens; FL1 is a 24,000 feedlot with two hospital-pens; and FL2 is a 24,000 cattle feedlot with four hospital-pens (in all the layouts n = 200 cattle per home-pen). Results for FS1 (4,000-cattle feedlot with one hospital-pen) are not shown because the interventions were never successful*.

d *Depopulation rates modeled*.

e *FM1 has 1 hospital-pen, FM2 has two hospital-pens, FL1 has two hospital-pens, and FL2 has four hospital-pens*.

f *FMD detection occurred when the proportion of clinical cattle in the index home-pen reached a 3% prevalence threshold*.

## Discussion

To our knowledge, our model is the first to describe the application of on-farm intervention strategies in the face of a potential FMD outbreak in U.S. beef feedlots. Based on our knowledge of the feedlot production system and previous experience of FMD epidemics in non-endemic countries, we evaluated the impact of movement restriction within the feedlot during the outbreak, and two partial depopulations strategies combined with movement restrictions on outbreak progression.

The interventions modeled had no effect on the projected duration of the outbreak and eventual infection of the entire feedlot (NH) or the number of remaining uninfected home-pens (NH-BD and NH-TD) in small sized FS1 feedlots. While feedlots with one-time head capacity of 4,000 or less represent ~27% of the cattle on-feed population, they represent up to 97% of the feedlots in the country (17). This model suggests that partial or targeted depopulation may have little effect on disease in these feedlots. NH was found to significantly decrease the projected outbreak progression in feedlots that operated with more home-pens per hospital-pen such as FM1 and FL1 (60 home-pens per hospital-pen). In feedlots that operated with fewer home-pens per hospital-pen such as FM2 and FL2 (30 home-pens per hospital-pen), the projected outbreak progression was delayed by NH but not as much as for FM1 and FL1. However, it is important to emphasize that, though delayed, the entire population in these feedlots was still infected. NH may be a useful strategy in medium- and large-size feedlots to delay infection progression while preparing logistics for other intervention strategies such as vaccination, however this was not assessed by the current model. In reality, complete stoppage of mixing of cattle from different home-pens in a hospital system might not be feasible. Any attempt to do so would likely require treatment within home-pens or use of portable hospital facilities that could move between home-pens. The impact of increased entry into home-pens or the use of portable hospital-pens on transmission within the feedlot was not assessed in this model. Also, complementary interventions to improve the success of movement restrictions such as well-defined biocontainment practices should be developed in advance as suggested by Brandt et al. (35). Temporary movement restriction during the period of targeted depopulation is also not assessed in this model but could be implemented.

Implementation of NH-BD (NH combined with barrier depopulation) under our assumptions was partially effective on medium- (50% of home-pens uninfected after the intervention) and large-size feedlots (25% of home-pens uninfected after the intervention) when higher depopulation rates were implemented (1,000 or more cattle per day for medium-size feedlots, and 2,000 or more cattle per day for large-size feedlots). However, this was following a depopulation of 50% of home-pens. We used an inside-out strategy of depopulation which means that depopulation was conducted starting with the index home-pen and then home-pens surrounding the index home-pen. An outside-in strategy could be explored to assess if there is any advantage.

Implementation of NH-TD (NH combined with targeted depopulation) was poorly effective in all feedlots modeled. In FM1, the intervention was partially successful to prevent infection in up to 30% of home-pens when the depopulation rate was 1,000 or 2,000 cattle per day, although 65–70% of home-pens in the feedlot had to be depopulated. For FM2, ~35–50% of home-pens were depopulated, and for FL1 and FL2 between 20 and 30% were depopulated but only a few uninfected home-pens were present at the end of the outbreak in successful simulations. We highlight that for all strategies we modeled an optimistic day of FMD detection of 3% clinical animals in the index home-pen by observational surveillance of pen-riders. While pen-riders are experienced personnel in detecting diseased animals (36, 37), clinical signs of FMD are very similar to other diseases which may confuse detection (38). Initial clinical detection will be followed by laboratory confirmation which can take up to several days depending on the logistics to collect and ship samples, and conduct the required tests to confirm FMDv suspicion as discussed by Sutmoller et al. (39) in their description of FMD outbreaks in the early 2000s. However, another study conducted by Walz et al. (40) modeled a detection threshold of 5% prevalence of clinical animals in beef herds with 5,000–50,000 one-time head capacity and conducted a sensitivity analysis testing 2.5 and 10% detection thresholds and found that the time-to-detection was not sensitive to those changes. Other methods for early detection such as the use of a surveillance test are not currently available but could be explored in the future. Other authors have discussed the potential use of real-time polymerase chain reaction (Rt-PCR) to test the saliva of animal in ropes in pens as a surveillance method to detect FMDv during the pre-clinical stage (41). Our model suggests that even with optimistic early detection of FMD within the feedlot, modeled methods are not sufficient to reliably stop an outbreak. Since interventions following our early detection were not sufficient, we did not model later detection times.

Depopulation strategies in previous outbreaks in Ireland, the Netherlands, and the UK attempted depopulation of affected cattle and/or susceptible cattle within 2 days (19, 42, 43). This might be feasible for these countries where the average herd size is <100 cattle (44); however, the experience in the UK demonstrated that the implementation of this policy was difficult to achieve (3, 45, 46). A requirement to complete depopulation on a large feedlot in 2 days is likely unrealistic even for the partial depopulations modeled by the current model. McReynolds and Sanderson (27) conducted a survey to investigate the feasibility of depopulation in large feedlots during a health emergency event such as an FMD outbreak and concluded that the methods explored were not viable to ensure a rapid, safe and humane depopulation.

We used a base depopulation rate of 1,000 cattle per day but also modeled higher depopulation rates than 1,000 cattle per day. We found that higher depopulation rates did not result in substantial differences in projected modeled outcomes compared to the base depopulation rate. Implementing higher depopulation rates than 1,000 cattle per day could be difficult to achieve, depending on available facilities. Hence, larger daily culling capacities modeled here may be optimistic. Moreover, facilities available in feedlots to dispose of depopulated carcasses might play a large role to limit rapid depopulation, however this limitation was not assessed in the current model. The cost of implementation should be explored in the future to make a more informed decision about the feasibility of implementation of partial or targeted depopulation strategies.

Another important factor to consider in our model is that for NH-TD we used a 100% accurate traceback prior to implementation of the depopulation strategies. Even good record-keeping in the feedlot likely will not achieve this level of trace-back accuracy. Since this level of accuracy was not effective, we did not explore less accurate traceback. There is only one report, to our knowledge, that addresses intervention strategies in a large feedlot (>14,000 cattle capacity) in South Africa (47). The authors reported that they adopted vaccination instead of depopulation due to the difficulties in maintaining bio-security measures during the depopulation of large number of animals. Other authors have suggested the potential implementation of selective depopulation which requires the culling of affected cattle based on the presence of clinical signs (27); however, the proportion of cattle to develop clinical FMD in a totally naïve population should be expected to be very high. An expert survey of FMD related parameters and clinical manifestation suggested that ~65–80% of cattle in U.S. beef feedlots might develop clinical FMD if infected by a high or low strain virulent strain, respectively (48).

In the sensitivity analysis, we found that the NH models were sensitive to changes in the latent and infectious periods. This suggests that introduction of high or low virulence strains could have an effect in the projected outbreak progression within the feedlots modeled. However, for NH-BD and NH-TD models, the duration of latent, infectious, and subclinical stages were less influential and other parameters had a larger effect on the projected outputs presented. For NH-BD, the projected day of FMD detection was the most influential parameters on probability of success in all feedlots modeled. This is not surprising since increased time to detection results in more time for spread between home-pens prior to implementation of interventions. However, for NH-TD, longer times for FMD detection were found to be associated with a higher likelihood to having uninfected home-pens. This can be explained by the fact that more home-pens have had contact with the hospital-pen in simulations with longer times to FMD detection, and therefore having uninfected home-pens after NH-TD might have been confounded by the number of home-pens depopulated by the intervention. For future models, different values for sensitivity of the observational surveillance, and delay in implementation of intervention due to FMD laboratory confirmation should be explored although we found that modeling an optimistic FMD detection threshold was already too late for the interventions to be highly successful. Finally, this model does not assess the potential effectiveness of vaccination and/or its combination with the intervention strategies modeled as a control option, and future work should evaluate the feasibility to implement vaccination.

## Conclusions

We believe we have captured the important structure and management aspects of U.S. feedlot systems and best estimates of FMD transmission parameters. Even with some optimistic assumptions, the three intervention strategies modeled were not highly effective in controlling the outbreak or required depopulation of a large proportion of cattle. Still, the results of our model should be interpreted with caution. Little data is available to inform the biological behavior of FMD in an immunologically naïve cattle population in confined production systems. Refinement of the methods used to model shedding and transmission along with better quality of data is needed to produce more robust models. The strategies should also be measured with a financial component to evaluate the cost-effectiveness of their implementation. Restriction of cattle movements from home-pens to hospital-pen proved to considerably prolong the outbreak in larger feedlots. Strategies combining vaccination with such movement restriction or targeted depopulation should be investigated. Finally, the exploration of different intervention strategies is challenging in beef feedlots in the U.S. because there are few other countries in the world with a similar production system and the immunologically naïve cattle population, so there is substantial uncertainty in how severe an FMD outbreak will be if the virus is introduced into the country.

## Data Availability Statement

The datasets generated for this study are available on request to the corresponding author.

## Author's Note

This work was adapted from a doctoral dissertation chapter available at: https://krex.k-state.edu/dspace/handle/2097/40307.

## Author Contributions

MS and VV conceived and designed the study. AC implemented the models and performed the sensitivity analyses. All authors contributed to the development, implementation, and analysis of the models and the output interpretation. All authors wrote the manuscript, read, and approved the final version for publication.

## Conflict of Interest

The authors declare that the research was conducted in the absence of any commercial or financial relationships that could be construed as a potential conflict of interest.
