# Supplementary material for: Modeling Intervention Scenarios During Potential Foot-and-Mouth Disease Outbreaks Within U.S. Beef Feedlots
Source: Front Vet Sci. 2021 Feb 16;8:559785. doi: 10.3389/fvets.2021.559785 (PMC7921729; doi:10.3389/fvets.2021.559785)
Supplement: Supplementary file 1 [file Data_Sheet_1.docx]

**SUPPLEMENTARY MATERIALS**


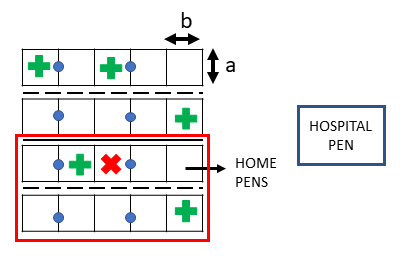


**Figure S 1 -** FS1 small-size feedlot with 4,000 cattle distributed in 20 pens (200 cattle per home-pen), and one hospital-pen. Solid black lines – the drovers alley, dashed black lines – the feed alley, a – the width of the home-pens (61 meters), b – the length of the home-pens (75.2 meters), blue circle – the water troughs, red X – the index home-pen, red rectangle – the area of the feedlot that was depopulated in scenario NH-BD (depopulation of the row of home-pens containing the index home-pen and the row on one side of the index home-pen), green crosses show representative traced-back home-pens that had contact with hospital-pen up to 7 days before detection depopulated in scenario NH-TD.


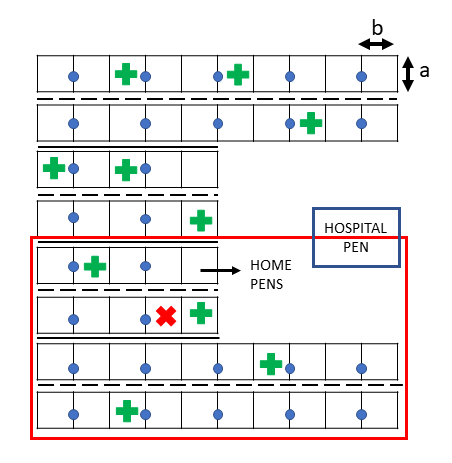


**Figure S 2 -** FM1 medium-size feedlot with 12,000 cattle distributed in 60 pens (200 cattle per pen), and one hospital-pen. Solid black lines – the drovers alley, dashed black lines – the feed alley, a – the width of the home-pens (61 meters), b – the length of the home-pens (75.2 meters), blue circle – the water troughs, red X – the index home-pen, red rectangle – the area of the feedlot that was depopulated in scenario NH-BD (depopulation of the row of home-pens containing the index home-pen, one row on the upper side of the index home-pen, and two rows on the lower side on the index home-pen), green crosses show representative traced-back home-pens that had contact with hospital-pen up to 7 days before detection depopulated in scenario NH-TD.


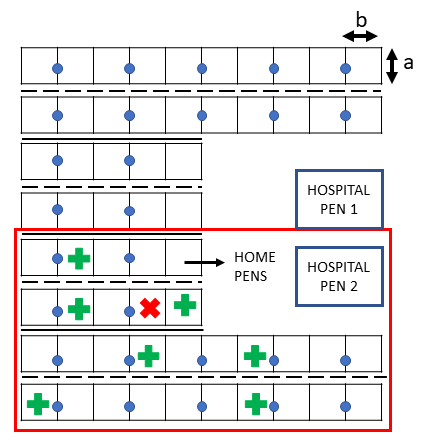


**Figure S 3 -** FM2 medium-size feedlot with 12,000 cattle distributed in 60 pens (200 cattle per pen), and two hospital-pens. Solid black lines – the drovers alley, dashed black lines – the feed alley, a – the width of the home-pens (61 meters), b – the length of the home-pens (75.2 meters), blue circle – the water troughs, red X – the index home-pen, red rectangle – the area of the feedlot that was depopulated in scenario NH-BD (depopulation of the row of home-pens containing the index home-pen, one row on the upper side of the index home-pen, and two rows on the lower side on the index home-pen), green crosses show representative traced-back home-pens that had contact with hospital-pen up to 7 days before detection depopulated in scenario NH-TD.


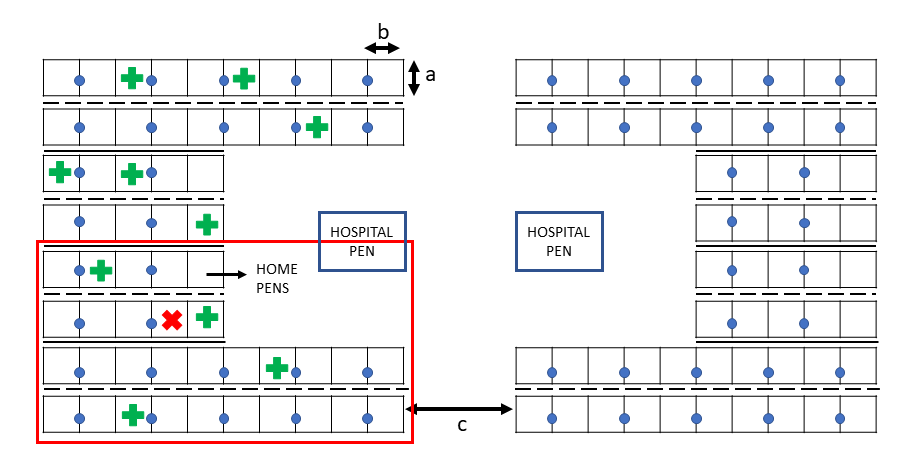


**Figure S 4 -** FL1 large-size feedlot with 24,000 cattle distributed in 120 pens (200 cattle per pen), and one hospital-pen. Solid black lines – the drovers alley, dashed black lines – the feed alley, a – the width of the home-pens (61 meters), b – the length of the home-pens (75.2 meters), c – the distance between section of home-pens (30 meters), blue circle – the water troughs, red X – the index home-pen, red rectangle – the area of the feedlot that was depopulated in scenario NH-BD (depopulation of the row of home-pens containing the index home-pen, one row on the upper side of the index home-pen, and two rows on the lower side on the index home-pen), green crosses show representative traced-back home-pens that had contact with hospital-pen up to 7 days before detection depopulated in scenario NH-TD.


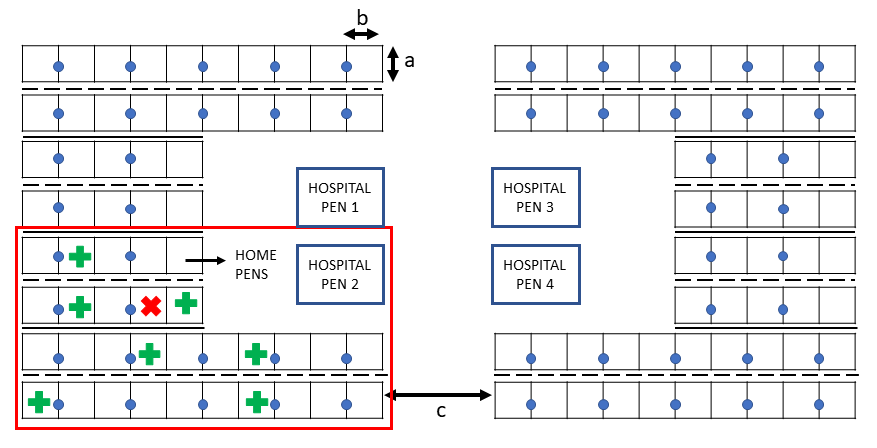


**Figure S 5 -** FL2 large-size feedlot with 24,000 cattle distributed in 120 pens (200 cattle per pen), and four hospital-pens. Solid black lines – the drovers alley, dashed black lines – the feed alley, a – the width of the home-pens (61 meters), b – the length of the home-pens (75.2 meters), c – the distance between section of home-pens (30 meters), blue circle – the water troughs, red X – the index home-pen, red rectangle – the area of the feedlot that was depopulated in scenario NH-BD (depopulation of the row of home-pens containing the index home-pen, one row on the upper side of the index home-pen, and two rows on the lower side on the index home-pen), green crosses show representative traced-back home-pens that had contact with hospital-pen up to 7 days before detection depopulated in scenario NH-TD.
